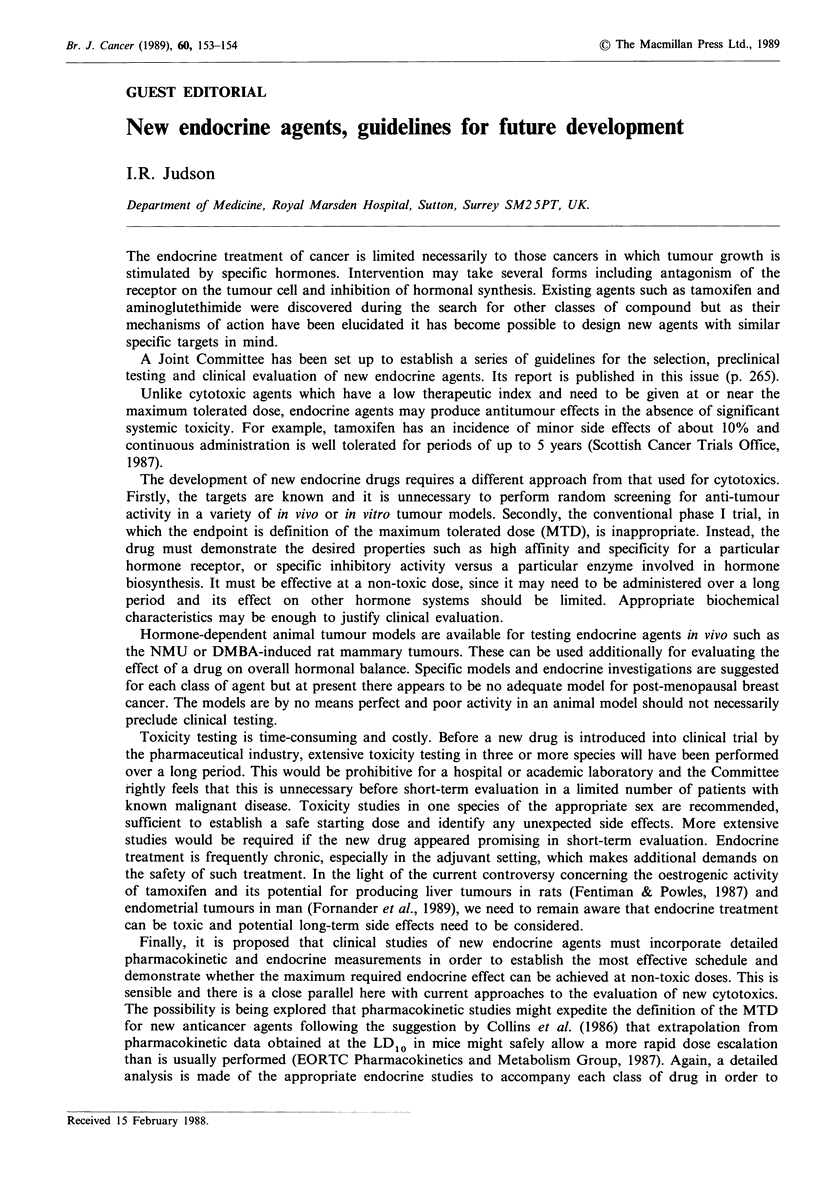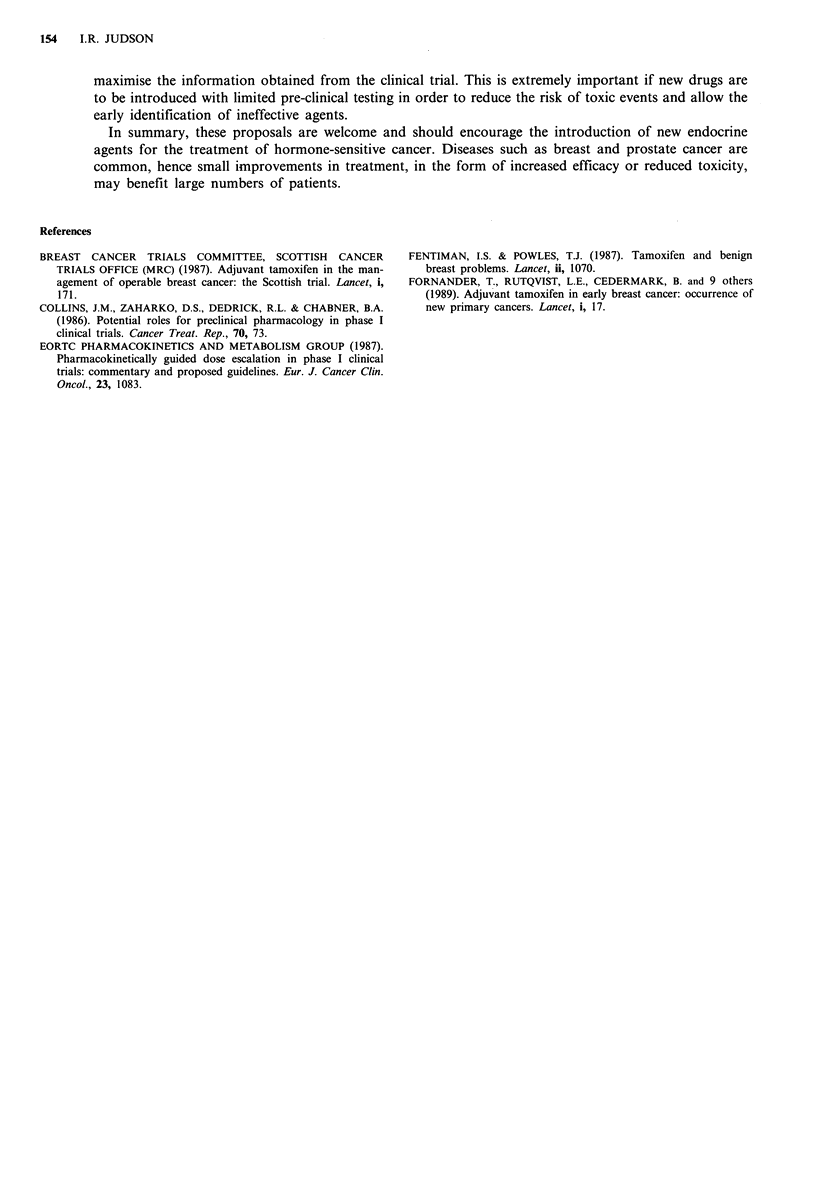# New endocrine agents, guidelines for future development.

**DOI:** 10.1038/bjc.1989.240

**Published:** 1989-08

**Authors:** I. R. Judson

**Affiliations:** Department of Medicine, Royal Marsden Hospital, Sutton, Surrey, UK.


 © The Macmillan Press Ltd., 1989

GUEST EDITORIAL

### New endocrine agents, guidelines for future development

I.R. Judson

*Department of Medicine, Royal Marsden Hospital, Sutton, Surrey SM2 5PT, UK.*

The endocrine treatment of cancer is limited necessarily to those cancers in which tumour growth is stimulated by specific hormones. Intervention may take several forms including antagonism of the receptor on the tumour cell and inhibition of hormonal synthesis. Existing agents such as tamoxifen and aminoglutethimide were discovered during the search for other classes of compound but as their mechanisms of action have been elucidated it has become possible to design new agents with similar specific targets in mind.

A Joint Committee has been set up to establish a series of guidelines for the selection, preclinical testing and clinical evaluation of new endocrine agents. Its report is published in this issue (p. 265).

Unlike cytotoxic agents which have a low therapeutic index and need to be given at or near the maximum tolerated dose, endocrine agents may produce antitumour effects in the absence of significant systemic toxicity. For example, tamoxifen has an incidence of minor side effects of about 10% and continuous administration is well tolerated for periods of up to 5 years (Scottish Cancer Trials Office, 1987).

The development of new endocrine drugs requires a different approach from that used for cytotoxics. Firstly, the targets are known and it is unnecessary to perform random screening for anti-tumour activity in a variety of *in vivo* or *in vitro* tumour models. Secondly, the conventional phase I trial, in which the endpoint is definition of the maximum tolerated dose (MTD), is inappropriate. Instead, the drug must demonstrate the desired properties such as high affinity and specificity for a particular hormone receptor, or specific inhibitory activity versus a particular enzyme involved in hormone biosynthesis. It must be effective at a non-toxic dose, since it may need to be administered over a long period and its effect on other hormone systems should be limited. Appropriate biochemical characteristics may be enough to justify clinical evaluation.

Hormone-dependent animal tumour models are available for testing endocrine agents *in vivo* such as the NMU or DMBA-induced rat mammary tumours. These can be used additionally for evaluating the effect of a drug on overall hormonal balance. Specific models and endocrine investigations are suggested for each class of agent but at present there appears to be no adequate model for post-menopausal breast cancer. The models are by no means perfect and poor activity in an animal model should not necessarily preclude clinical testing.

Toxicity testing is time-consuming and costly. Before a new drug is introduced into clinical trial by the pharmaceutical industry, extensive toxicity testing in three or more species will have been performed over a long period. This would be prohibitive for a hospital or academic laboratory and the Committee rightly feels that this is unnecessary before short-term evaluation in a limited number of patients with known malignant disease. Toxicity studies in one species of the appropriate sex are recommended, sufficient to establish a safe starting dose and identify any unexpected side effects. More extensive studies would be required if the new drug appeared promising in short-term evaluation. Endocrine treatment is frequently chronic, especially in the adjuvant setting, which makes additional demands on the safety of such treatment. In the light of the current controversy concerning the oestrogenic activity of tamoxifen and its potential for producing liver tumours in rats (Fentiman & Powles, 1987) and endometrial tumours in man (Fornander *et al.*, 1989), we need to remain aware that endocrine treatment can be toxic and potential long-term side effects need to be considered.

Finally, it is proposed that clinical studies of new endocrine agents must incorporate detailed pharmacokinetic and endocrine measurements in order to establish the most effective schedule and demonstrate whether the maximum required endocrine effect can be achieved at non-toxic doses. This is sensible and there is a close parallel here with current approaches to the evaluation of new cytotoxics. The possibility is being explored that pharmacokinetic studies might expedite the definition of the MTD for new anticancer agents following the suggestion by Collins *et al.* (1986) that extrapolation from pharmacokinetic data obtained at the $LD_{10}$ in mice might safely allow a more rapid dose escalation than is usually performed (EORTC Pharmacokinetics and Metabolism Group, 1987). Again, a detailed analysis is made of the appropriate endocrine studies to accompany each class of drug in order to

Received 15 February 1988.

maximise the information obtained from the clinical trial. This is extremely important if new drugs are to be introduced with limited pre-clinical testing in order to reduce the risk of toxic events and allow the early identification of ineffective agents.

In summary, these proposals are welcome and should encourage the introduction of new endocrine agents for the treatment of hormone-sensitive cancer. Diseases such as breast and prostate cancer are common, hence small improvements in treatment, in the form of increased efficacy or reduced toxicity, may benefit large numbers of patients.

### References

BREAST CANCER TRIALS COMMITTEE, SCOTTISH CANCER TRIALS OFFICE (MRC) (1987). Adjuvant tamoxifen in the management of operable breast cancer: the Scottish trial. *Lancet*, **i**, 171.

COLLINS, J.M., ZAHARKO, D.S., DEDRICK, R.L. & CHABNER, B.A. (1986). Potential roles for preclinical pharmacology in phase I clinical trials. *Cancer Treat. Rep.*, **70**, 73.

EORTC PHARMACOKINETICS AND METABOLISM GROUP (1987). Pharmacokinetically guided dose escalation in phase I clinical trials: commentary and proposed guidelines. *Eur. J. Cancer Clin. Oncol.*, **23**, 1083.

FENTIMAN, I.S. & POWLES, T.J. (1987). Tamoxifen and benign breast problems. *Lancet*, **ii**, 1070.

FORNANDER, T., RUTQVIST, L.E., CEDERMARK, B. and 9 others (1989). Adjuvant tamoxifen in early breast cancer: occurrence of new primary cancers. *Lancet*, **i**, 17.